# PTFE-Carbon Nanotubes and Lipase B from *Candida antarctica*—Long-Lasting Marriage for Ultra-Fast and Fully Selective Synthesis of Levulinate Esters

**DOI:** 10.3390/ma14061518

**Published:** 2021-03-19

**Authors:** Anna Szelwicka, Agnieszka Siewniak, Anna Kolanowska, Sławomir Boncel, Anna Chrobok

**Affiliations:** 1 Department of Chemical Organic Technology and Petrochemistry, Silesian University of Technology, Krzywoustego 4, 44-100 Gliwice, Poland; Anna.Szelwicka@polsl.pl (A.S.); Agnieszka.Siewniak@polsl.pl (A.S.); 2 Department of Organic Chemistry, Bioorganic Chemistry and Biotechnology, Krzywoustego 4, 44-100 Gliwice, Poland; Anna.Kolanowska@polsl.pl

**Keywords:** levulinate esters, levulinic acid, lipase, carbon nanotubes

## Abstract

An effective method for levulinic acid esters synthesis by the enzymatic Fischer esterification of levulinic acid using a lipase B from *Candida antarctica* (CALB) immobilized on the advanced material consisting of multi-wall carbon nanotubes (MWCNTs) and a hydrophobic polymer—polytetrafluoroethylene (Teflon, PTFE)—as a heterogeneous biocatalyst, was developed. An active phase of the biocatalyst was obtained by immobilization via interfacial activation on the surface of the hybrid material MWCNTs/PTFE (immobilization yield: 6%, activity of CALB: 5000 U∙L∙kg^−1^, enzyme loading: 22.5 wt.%). The catalytic activity of the obtained biocatalyst and the effects of the selected reaction parameters, including the agitation speed, the amount of PTFE in the CALB/MWCNT-PTFE biocatalyst, the amount of CALB/MWCNT-PTFE, the type of organic solvent, *n*-butanol excess, were tested in the esterification of levulinic acid by *n*-butanol. The results showed that the use of a two-fold excess of levulinic acid to *n*-butanol, 22.5 wt.% of CALB on MWCNT-PTFE (0.10 wt.%) and cyclohexane as a solvent at 20 °C allowed one to obtain *n*-butyl levulinate with a high yield (99%) and selectivity (>99%) after 45 min. The catalyst retained its activity and stability after three cycles, and then started to lose activity until dropping to a 69% yield of ester in the sixth reaction run. The presented method has opened the new possibilities for environmentally friendly synthesis of levulinate esters.

## 1. Introduction

Nowadays, upgrading the biomass-derived platform chemicals toward commodity and specialty chemicals is one of the central challenges in the development of sustainable biorefinery [1]. Levulinic acid (LA), as the final product of the thermal acid-catalysed hydrolysis of lignocellulose, is one of the important bio-based chemicals which can be used as building block for drugs, plasticizers, fragrances, and cosmetics. LA can be also transformed into a broad spectrum of higher value-added building blocks, such as levulinic acid esters (LAEs) [2,3]. LAEs are environmentally friendly compounds, do not cause mutagenicity or carcinogenicity, have significantly lower vapor pressures compared to the common solvents, and therefore are considered to be “green solvents”. LAE can be used as additives in diesel formulations and production of plasticizers (*n*-butyl, *n*-hexyl and cyclohexyl levulinates) as well as flavours and fragrances in the food and perfume industries [2,3]. LAEs can be obtained directly from polysaccharides at a high temperature (175 °C) [4] or from furfuryl alcohol at 120−140 °C, in the presence of sulfonated materials [4], or acidic ionic liquids [5]. Another known method is the catalytic one-pot upgrading of α-angelica lactone (5-methyl-2(3*H*)-furanone, α-AL) in the presence of an acidic catalyst [6,7,8] at 60 °C or via biotransformation at ambient temperature [9].

The fundamental way to obtain LAEs can be the direct esterification of LA with alcohols in the presence of acidic catalysts [10]. Esterification is a thermodynamically controlled process; thus, yields are related to thermodynamics of the reaction [11,12,13]. The formation of water as a by-product in a thermodynamically controlled process is the reason for non-quantitative atom economy [14]. Thus, this method suffers from problems connected with a need to shift the reaction equilibrium in the process. Biocatalysis brings a promising alternative for the synthesis of esters characterized by the unique advantages over the chemical catalysis. Enzymatic processes provide mild reaction conditions, higher selectivity and a limited amount of generated waste [15]. Nevertheless, the main problem to circumvent during the design of enzymatic processes is a poor stability of the protein beyond the natural environment [16]. Among known tools increasing the stability of an enzyme, immobilization on the solid support is the most promising from the industrial application point-of-view [17,18,19]. Immobilization allows one to improve not only the stability of enzymes, but also their activity, selectivity, specificity, and resistance to the presence of inhibitors or purity of an immobilized enzyme [20,21,22]. In addition, this approach provides the easy separation and recycling of biocatalysts, as well as the possibility to apply it in the flow system [23,24].

The physical immobilization of lipases on hydrophobic supports is an example of improving their stability and activity. The phenomenon named “interfacial activation” is responsible for the increase in the activity of the majority of lipases at the interface [25,26]. Lipases usually possess an externally hydrophilic lid, which covers the hydrophobic tunnel leading to the active centre. The lid opens at the water/oil interface, the active centre is more exposed, and the lipase turns into an active form as the consequence [27,28]. This phenomenon can be a reason for decreasing activity as well in the case of high concentrations of the lipase due to the tendency to form dimeric agglomerates [29,30,31]. Lipase B from *Candida antarctica* is one of the most commonly applied lipases due to the relatively small lid, unable to fully cover its hydrophobic pocket. Hence, its high stability is related to the limited formation of dimeric agglomerates [32,33].

Surprisingly, only a few papers describe the use of enzymes (mostly immobilized lipase B from *Candida antarctica*, CALB) for the esterification of LA. Two methods for CALB anchoring on the surface of solid carriers were used. Covalent immobilization can prevent the enzyme from leaching and help the enzyme to maintain its stability. Unfortunately, it also influences the economy of the process by adding the extra step in the biocatalyst preparation [34,35,36,37]. The first studies were carried out in the presence of commercially available Novozym 435 (CALB immobilized on acrylic resin, Lewatit VP OC 1600) [34]. The synthesis of *n*-butyl levulinate by the esterification of levulinic acid with *n*-butanol (molar ratio 1:2) at 50 °C, with *tert*-butyl methyl ether as the solvent, yielded 80% of *n*-butyl levulinate. The recycling of the Novozyme-435 was not presented in this work [34]. Actually, the above commercial biocatalyst was previously verified as not promising for recycling. This resin could swell upon contact with the organic solvent, and the enzyme would inevitably lose its activity [9]. The next trials were carried out with CALB immobilized on organosilica material with highly ordered 3D macroporous organosilica frameworks by simple adsorption [35]. The yield of ester reached 75% at 40 °C after 12 h using a molar ratio of acid to alcohol 1:10. After nine cycles of the reaction, the yield of ester dropped to 50%. The covalent immobilization of CALB on the hydrophobic organosilica nanoparticles was also demonstrated [36]. Esterification of LA and *n*-lauryl alcohol (50 °C; LA:*n*-lauryl alcohol, 1:11.5; 22.6 h) achieved the 76% conversion of LA which dropped to 30% in nine cycles of the reaction. The application of enzymes other than CALB resulted in even lower conversions of LA. So, lipase LipR2 from *Alkalispirillum sp.* covalently immobilized on Florisil^®^ in the esterification of levulinic acid with *n*-butanol (molar ratio 1:10) at 55 °C yielded 46% of ester after 12 h [37]. Moreover, the stability of the gradually dropped through nine reaction cycles to 48% of its initial activity.

The scrupulous analysis of the up-to-date literature data—revealing medium yields of esters accompanied by rather moderate stability of the biocatalytic systems and extended esterification times—encouraged us to demonstrate the potential of the active solid carrier for CALB. In our previous studies, a highly active nanobiocatalyst from CALB non-covalently immobilized on multi-walled carbon nanotubes (MWCNTs) for the Baeyer–Villiger synthesis of lactones was presented [9,38,39,40]. The direct physical adsorption method was based on hydrophobic, electrostatic and hydrogen bonding interactions between MWCNTs and the enzyme. A particularly efficient upgrade of the process was achieved by transfer from a batch to a flow system [39]. Again, high activity and the enhanced stability of the same biocatalyst in the esterification of dicarboxylic acids for the production of diester plasticizers was further verified [40].

Recent interest in the application of nanomaterials as carriers for enzymes caused by, i.e., the large and designable surface area and high volume/size ratio, make them excellent materials for immobilization [41,42,43]. The idea of using hydrophobic MWCNTs for the designing of biocatalysts has been evolving. Significant progress has been obtained by the modification of MWCNTs with small amounts of polytetrafluoroethylene (PTFE, 0.10 wt.%) as hydro- and oleophobic microparticles [9]. The hybrid carrier was used for the CALB immobilization via interfacial activation [9]. This amalgamation allowed for the synergetic enhancement of the properties of the biocatalytic system toward its increased stability and superactivity. The catalytic system was used for the first time in the enzymatic route for the synthesis of alkyl levulinates from α-angelica lactone. The unique stability of the developed biocatalyst was demonstrated in six and three reaction cycles at 20 °C and 60 °C, respectively.

Herein, we present the development in biocatalytic transformations using the advanced material (MWCNTs/PTFE) as a hybrid platform for CALB immobilization. The presented new concept of the LA esterification with a variety of alcohols leads to more environmentally benign processes for the synthesis of alkyl levulinates encompassing superactivity, full selectivity, stability and recyclability.

## 2. Materials and Methods

### 2.1. Materials

Alcohols (purity min. 99%), solvents (purity min. 99%), *n*-decane (purity 99%) (internal standard), levulinic acid (purity 99.8 %), native lipase B from *Candida antarctica* (activity 5000 U∙L∙kg^−1^), native lipase from *Candida rugosa* (activity 700 U∙L∙kg^−1^), native lipase from *Aspergillus oryzae* (activity 20,000 U∙L∙kg^−1^) and polytetrafluoroethylene (6–9 μm grains, Teflon, PTFE^®^) were purchased from Sigma–Aldrich (St. Louis, MO, USA). Industrial grade MWCNTs (outer diameter = 20–40 nm, length = 10–30 μm) were purchased from Cheap Tubes Inc. (Grafton, VT, USA).

### 2.2. GC-FID Analyses

GC-FID analyses were performed on a Shimadzu GC-2010 Plus equipped with a Zebron ZB 5MSi column (30 m × 0.32 mm × 0.25 µm film). Products were analysed by NMR spectroscopy (Varian NMR system, ^1^H NMR spectra at 600 MHz and ^13^C NMR at 151 MHz). See ESI, Chapter S2.

### 2.3. Thermogravimetric Analyses (TGA)

Thermogravimetric analyses (TGA) were conducted using a LINSEIS STA PT1600 (Selb, Germany). The samples (20–40 mg) were heated in alumina crucibles up to 800 °C in Ar flowing atmosphere (50 sccm) for a heating rate of 10 °C/min.

### 2.4. Transmission Electron Microscopy (TEM)

Transmission electron microscopy (TEM) images were performed on a Tecnai F20 TWIN microscope (FEI Company, Norcross, GAUSA) equipped with a field emission gun, setting an acceleration voltage at a value of 200 kV. The Eagle 4k HS camera (FEI Company, USA) was applied for recording images. The obtained images were processed with TIA software (FEI Company, USA) (ESI, Chapter S1, Figures S1 and S2). A copper grid with holey carbon film was used for the preparing samples.

### 2.5. Raman Spectra

Raman spectra were obtained using a Raman system (Renishaw, Germany) at 633 nm (a red laser) with the laser power of 5%, 20× magnification and an exposure time of 10 s. For each material, three accumulations were collected in three locations of the sample.

### 2.6. Synthesis of Hybrid MWCNT-PTFE Support

In a 400-mL beaker, demineralized water (150 mL), industrial grade MWCNTs (5.00 g), micro-size grains polytetrafluoroethylene (0.05–0.05 g, 0.01–10.00 wt.% of MWCNTs) and Pluronic F-127 (non-ionic triblock copolymer of polyethylene glycol and polypropylene glycol) (0.05 g) were homogenized using Silverson L5M-A laboratory mixer. After 30 min (4500 rpm) the hybrid MWCNT-PTFE support was filtered off, washed with water (20 mL) and dried under reduced pressure at a Schlenk line (1 mbar, 30 °C, 24 h).

### 2.7. Immobilization of Lipases 

Immobilization was performed according to a literature method [9,38,40]. A native lipase (7.5 g), a hybrid MWCNT-PTFE support (1.0 g) and water (30 mL) (pH = 6.20–6.30) was introduced into a 100-mL round bottom flask and shaken in a thermostatic shaker (250 rpm) at 20 °C for 3 h. Then, the solid was filtered off under vacuum and washed 4 times with water (50 mL). The obtained catalyst was dried over anhydrous P_2_O_5_ at 5 °C for 3 days.

### 2.8. Esterification Reaction

A biocatalyst (25–200 mg), an organic solvent (0–1 mL/1 mmol LA), levulinic acid (0.5 mmol), alcohol (0.5–11 mmol) and *n*-decane as the internal standard (20 wt.% relative to LA), were added in this order to a two-neck round-bottom flask (10 mL). The flask was placed in a thermostatic shaker (100–250 rpm) and the reaction was carried out at 25 °C for 40–120 min. During the reaction, samples (10 µL) were taken from reaction mixture, diluted with 1.5 mL of acetonitrile and analysed by GC-FID. After completion of the reaction, the biocatalyst was filtered off and washed with cyclohexane (20 mL).

### 2.9. The Product Isolation

After completion of the reaction, the biocatalyst was filtered off. Then, the solvent was evaporated from the filtrate. If the boiling point of the product exceeded 200 °C, vacuum distillation was used to purify the ester. Isopropyl levulinate which can undergo partial hydrolysis at elevated temperatures was further purified by column chromatography. Ethyl acetate:cyclohexane (2:5 = *v/v*) was used as a mobile phase and Al_2_O_3_ as the stationary phase. The full signal assignments for ^1^H and ^13^C NMR spectra of products were presented in ESI (ESI, Chapter S3).

### 2.10. ^1^H and ^13^C NMR Spectra

Ethyl levulinate: ^1^H NMR (600 MHz, CDCl_3_, TMS), δ (ppm): 1.26 (t, J = 7.1 Hz, 3H), 2.20 (s, 3H), 2.57 (t, J = 6.6 Hz, 2H), 2.75 (t, J = 6.6 Hz, 2H), 4.13 (q, J = 7.1 Hz, 2H). ^13^C NMR (151 MHz, CDCl_3_), δ (ppm): 14.28, 28.15, 29.99, 38.08, 60.74, 172.86, 206.79.

*n*-Propyl levulinate: ^1^H NMR (600 MHz, CDCl_3_, TMS), δ (ppm): 0.94 (t, J = 7.4 Hz, 3H), 1.65 (dd, J = 14.2, 6.9 Hz, 2H), 2.19 (s, 3H), 2.58 (t, J = 6.6 Hz, 2H), 2.75 (t, J = 6.6 Hz, 2H), 4.04 (t, J = 7.6 Hz, 2H); ^13^C NMR (151 MHz, CDCl_3_), δ (ppm): 0.94 (t, J = 7.4 Hz, 3H), 1.65 (dd, J = 14.2, 6.9 Hz, 2H), 2.19 (s, 3H), 2.58 (t, J = 6.6 Hz, 2H), 2.75 (t, J = 6.6 Hz, 2H), 4.04 (t, J = 7.6 Hz, 2H).

Isopropyl levulinate: ^1^H NMR (600 MHz, CDCl_3_, TMS), δ (ppm): 1.14 (d, J = 6.3 Hz, 6H), 2.11 (s, 3H), 2.46 (t, J = 6.6 Hz, 2H), 2.65 (t, J = 6.7 Hz, 2H), 4.91 (hept, J = 6.3 Hz, 1H). ^13^C NMR (151 MHz, CDCl_3_), δ (ppm): 21.69, 28.30, 29.80, 37.92, 67.91, 172.17, 206.68.

*n*-Butyl levulinate: ^1^H NMR (600 MHz, CDCl_3_, TMS): δ(ppm): 0.90 (t, J = 7.4 Hz, 3H), 1.34 (dd, J = 15.0, 7.5 Hz, 2H), 1.51–1.63 (m, 2H), 2.16 (s, 3H), 2.54 (t, J = 6.6 Hz, 2H), 2.71 (t, J = 6.6 Hz, 2H), 4.04 (t, J = 6.7 Hz, 2H). ^13^C NMR (151 MHz, CDCl_3_), δ (ppm): 13.80, 19.22, 28.13, 29.98, 30.74, 38.09, 64.67, 172.94, 206.78.

*n*-Dodecyl levulinate: ^1^H NMR (600 MHz, CDCl_3_, TMS), δ (ppm): 0.77–0.89 (m, 3H), 1.25 (m, 18 H), 1.61 (m, 2H), 2.18 (m, 3H), 2.57 (dd, J = 6.2, 3.1 Hz, 2H), 2.74 (dd, J = 6.1, 3.0 Hz, 2H), 3.99–4.08 (m, 2H). ^13^C NMR (151 MHz, CDCl_3_), δ (ppm): 14.25, 22.83, 26.03, 28.16, 28.73, 29.39, 29.48, 29.66, 29.71, 29.78, 30.02, 32.06, 38.12, 65.02, 172.95, 206.72.

### 2.11. Catalyst Recycling

After the reaction, the immobilized catalyst was separated from the mixture by filtration, washed with cyclohexane (20 mL,) dried under vacuum (1 mbar, 20 °C, 4 h) and used for the next cycle.

## 3. Results

The hybrid support based on MWCNTs modified with PTFE was used in our previous work for the immobilization of CALB [9]. It is known from the literature [44,45,46,47] that hydrophobic supports used for lipase immobilization via interfacial activation influences its activity and stability. In order to increase the hydrophobicity of MWCNTs, small initial amounts such as 0.1 wt.% of PTFE was determined as the most influential—in light of the overall catalytic performance—surface content [9]. The final biocatalyst was obtained by the adsorption of CALB (22.5 wt.%, Table 1) and characterized via microscopic, thermogravimetric and adsorption techniques. In this work, additional Raman spectroscopy analysis for the MWCNT-PTFE (0.1 wt.%) support, PTFE and the CALB/MWCNT-PTFE (0.1 wt.%) biocatalyst were performed (Figure S3). Raman spectrum of the PTFE sample covers the main peaks at 253 cm^−1^, 343 and 359 cm^−1^ which correspond to the torsional and deformational vibrations of the CF_2_ groups. In turn, the band at 732 cm^−1^ is related to symmetric CF_2_-stretching. Bands at 1302 cm^−1^ and 1381 cm^−1^ can be assigned to the C-C stretching. For the MWCNT-PTFE (0.1 wt.%) support, three Raman features are observed. At 1586 cm^−1^, graphitization mode (G) connected to the in-plane bond stretching of the C-C bonds in the hexagonal lattice can be found. The G-band is a characteristic parameter for all of the sp^2^ carbon materials. Due to an elastic scattering of a photo-excited electron by crystal defects, the peak at 1334 cm^−1^ is observed (D-band). The band intensity ratio (I_D_/I_G_) is a parameter which can be correlated with the wall defects. For this sample, I_D_/I_G_ equals 1.56, which proves a rather significant number of crystallographic wall defects. For the CALB/MWCNT-PTFE (0.1 wt.%) biocatalyst Raman spectrum is similar to pristine CNTs. The amount of PTFE and CALB compared to the CNT matrix is small, hence it disappears in the noise. However, a finely developed peak at 734 cm^−1^ corresponding to the symmetric stretching vibrations of CF_2_ is clearly visible.

The high activity and stability of the biocatalyst in the synthesis of LAEs from biomass-derived α- angelica lactone in a comprehensive approach was presented. It should also be noted that the other role of PTFE was the protection of the enzyme macromolecules against agglomeration. The amount of PTFE was found as the decisive factor affecting both the CALB loading efficiency and further activity of the immobilized enzyme in the model reaction [9].

The immobilization step was employed according to the literature [18]. The immobilization yield was determined based on the lipase content in an aqueous glycerine solution (50 wt.%) and on the CALB loading in the biocatalysts, determined previously via the thermogravimetric method. The results were presented in Table 1. In addition, experiments were performed toward the determination of how the immobilization environment affected the activity of CALB and how significant the drop in activity of CALB in the filtrate after immobilization was. For this purpose, the following experiments were performed: (I) a reaction with the filtrate after the synthesis of the CALB/MWCNT-PTFE (0.1 wt.%) biocatalyst in an amount equal to the amount of CALB immobilized on the biocatalyst; (II) a reaction with the equivalent amount of aqueous solution of CALB after immobilization without support; and, as the reference, (III) a reaction with a fresh solution of CALB diluted by deionized water to the same amount as in experiments (I) and (II). These studies confirmed that the active particles of the biocatalyst were adsorbed on the support during the immobilization step, otherwise in a reaction with the filtrate (I) the lipase would have remained equally active to the CALB in reaction (II). In addition, a slight drop in an activity of CALB during the immobilization step was observed for the sake of a higher yield of *n*-butyl levulinate in the process (III). Nevertheless, further studies exhibited much higher activity of CALB immobilized on the MWCNT-PTFE supports than in any other case (Figure S4).

**Scheme 1:**
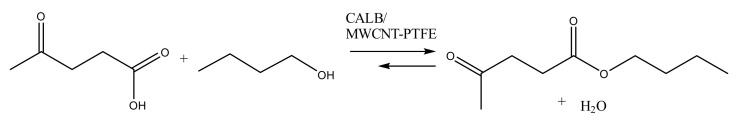
Enzymatic esterification of levulinic acid (LA) and *n*-butanol.

In this work, the high efficiency of the biocatalyst (Table 1) was cross-verified in the conventional esterification of LA with alcohols. Hence, in order to determine the favourable conditions of the model reaction between LA and *n*-butanol (Scheme 1), the influence of selected parameters, i.e., agitation speed, amount of PTFE in the CALB/MWCNT-PTFE system, amount of biocatalyst, type of organic solvent, LA:*n*-butanol molar ratio, and temperature was investigated.

The model reaction proceeded in a multi-phase system, with the organic phase consisting of substrates/product, solid biocatalyst and water. The initial studies determining the influence of the agitation speed on the reaction course were crucial for the elimination of the diffusion-driven kinetics. So, firstly, a two-fold molar excess of alcohol in relation to LA, *n*-hexane as the solvent and the biocatalyst CALB/MWCNT-PTFE (0.10 wt.%) were applied. The reactions were conducted in a thermostatic shaker at 20 °C. Due to the high tendency to promiscuous actions of CALB [48,49], the lipase is able to catalyse inter alia aldol condensation of compounds with a carbonyl group. LA contains one keto group, thus, it is essential to emphasise the highly selective actions of CALB in the model reaction and the legitimacy to present the value of selectivity towards the synthesis of *n*-butyl levulinate throughout.

**Figure 1:**
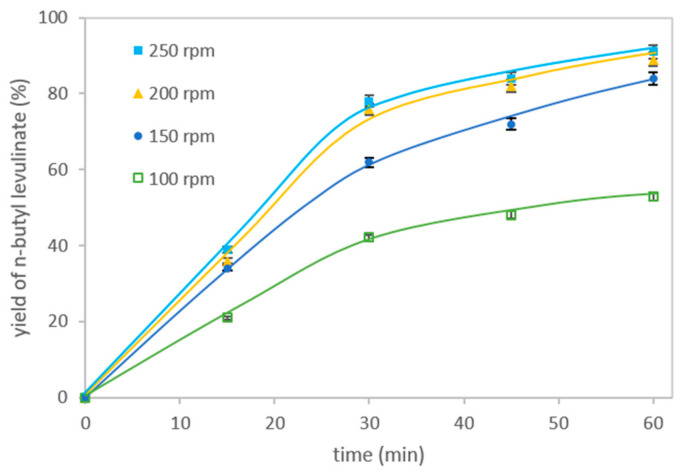
The effect of agitation speed on LA esterification. Reaction conditions: LA (0.117 g, 1 mmol); *n*-butanol (0.148 g, 2 mmol); biocatalyst CALB/MWCNT-PTFE (0.10 wt.%) (150 mg); *n*-hexane (1 mL); 20 °C.

The reaction rate increased with the agitation speed in the range from 100 rpm to 200 rpm (Figure 1). Above 200 rpm, the further increase in the stirring speed did not significantly affect the reaction kinetics. Nevertheless, to ensure that diffusion was not a limiting factor in the studied esterification, the subsequent experiments were performed at 250 rpm.

**Figure 2:**
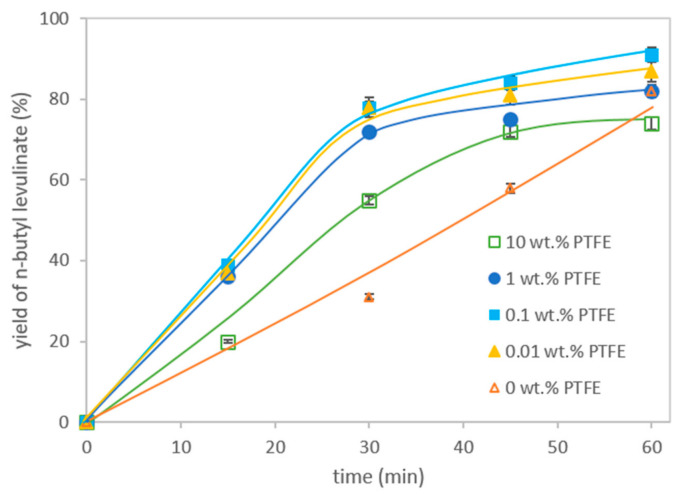
The effect of the amount of PTFE in CALB/MWCNT-PTFE catalyst on LA esterification. Reaction conditions: LA (0.117 g, 1 mmol); *n*-butanol (0.148 g, 2 mmol); CALB/MWCNT-PTFE (0.01–10.00 wt.%) biocatalyst (150 mg); *n*-hexane (1 mL); 20 °C; 250 rpm.

The effect of the optimal amount of PTFE additive for the activity of the biocatalyst was presented in Figure 2. The experiments were carried out in the presence of biocatalysts (Table 1), which consisted of CALB and the hybrid carrier modified with PTFE in the range from 0.01% to 10% by weight in relation to MWCNTs. The results clearly indicated that *n*-butyl levulinate was obtained in 87% and 91% yields, after 60 min, with CALB immobilized on the support with PTFE 0.01 wt.% and 0.1 wt.% content, respectively (Figure 2). Indeed, as shown, the optimum content of PTFE in MWCNTs (0.01–0.1 wt.%) provided the appropriate hydrophobic/hydrophilic balance of the catalyst, facilitating the access of the reactants to the lipase. Eventually, the latter CALB/MWCNT-PTFE (0.10 wt.%) system—as still slightly more efficient and also more controllable in handling—was selected for the further experiments.

**Figure 3:**
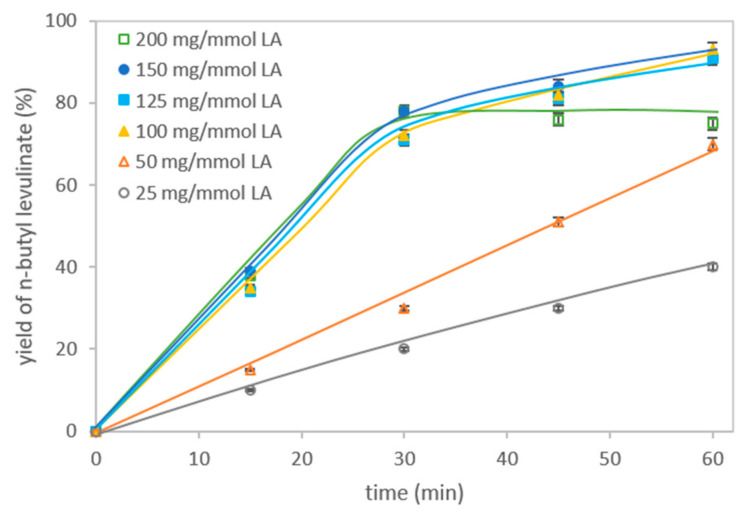
The effect of amount of CALB/MWCNT-PTFE on LA esterification. Reaction conditions: LA (0.117 g, 1 mmol); *n*-butanol (0.148 g, 2 mmol); biocatalyst CALB/MWCNT-PTFE (0.1 wt.%) (25–200 mg); *n*-hexane (1 mL); 20 °C; 250 rpm.

Further, the influence of the amount of the biocatalyst used on the yield of *n*-butyl levulinate was examined in the range from 25 to 200 mg/mmol of LA (Figure 3), clearly indicating that addition of 150 mg of the biocatalyst conforms the optimum content. In the case of the lower amount of the biocatalyst, the amount of an introduced lipase was insufficient for the substrates, thus the processes occurred with a slower reaction rate. However, a higher amount of the biocatalyst than 150 mg per 1 mmol of LA resulted in a difficulty in the mass transport caused by a huge amount of the solid particles in the reaction mixture. This led to the slowing down of the reaction and achieving a *plateau* (in Figure 3) after 30 min. As a consequence of these studies, 150 mg of the biocatalyst per 1 mmol of LA was used in further experiments as the favourable amount.

**Figure 4:**
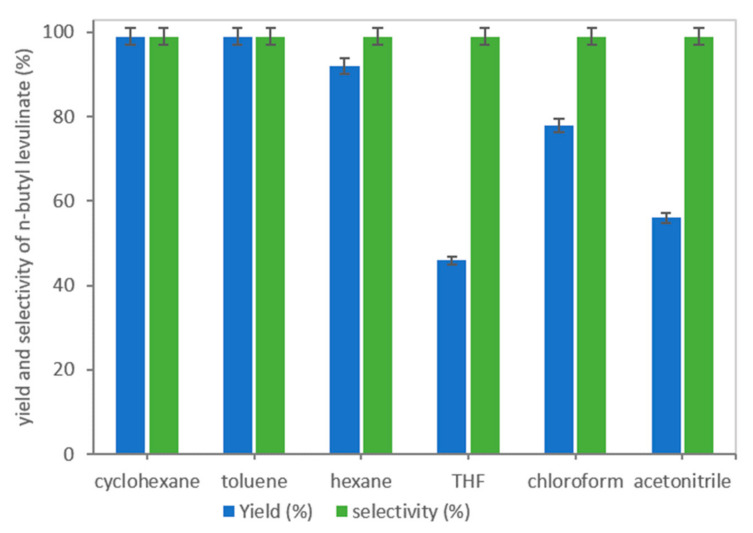
The effect of organic solvent on LA esterification. Reaction conditions: LA (0.117 g, 1 mmol); *n*-butanol (0.148 g, 2 mmol); biocatalyst CALB/MWCNT-PTFE (0.1 wt.%) (150 mg); solvent (1 mL); 20 °C; 250 rpm, 60 min.

Next, type of solvent—as the critical variable affecting the biocatalyst activity—was studied. According to the literature, lipases exhibit the highest activity in hydrophobic, aprotic solvents. This phenomenon is caused by the interfacial activation of the enzyme at the interface of the hydrophobic solvent and the water shell surrounding the protein [50]. Among the herein tested solvents, the most promising results were obtained for non-polar solvents such as cyclohexane, hexane and toluene, allowing for the yield of *n*-butyl levulinate almost quantitatively (Figure 4). For the use of polar solvents (THF, chloroform, acetonitrile) the yield of ester was drastically reduced. The multiple interactions of enzymes with the lone electron pairs of heteroatoms from the solvent molecules, as well as partial miscibility with water, could lead to conformational destabilization, a partial closure of the “lid”, blocking the access to the active site, and hence, finally, turning into the less active “closed” form.

**Figure 5:**
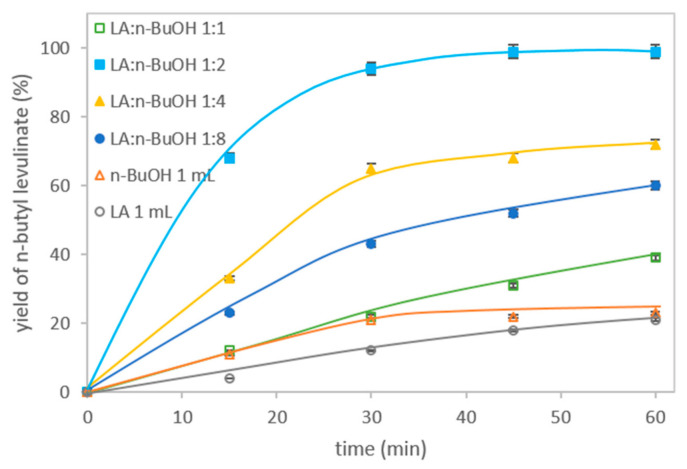
The effect of *n*-butanol excess on LA esterification. Reaction conditions: LA (0.117 g, 1 mmol or 1 mL of LA); *n*-butanol (1–11 mmol); CALB/MWCNT-PTFE (0.1 wt.%) biocatalyst (150 mg); cyclohexane (1 mL); 20 °C; 250 rpm.

Among the methods of shifting the equilibrium of reversible reactions towards the desired product, a use of an excess of one of the reactants (typically the inexpensive one) can be distinguished. In addition, the application of one of the substrates in an excess improves the kinetics of the process and allows one to overcome the equilibrium limitations. Simultaneously, the activity of an enzyme can be changed as well. Therefore, the effect of the excess of *n*-butanol on the course of the esterification reaction was also investigated. Figure 5 clearly shows that the use of a double molar excess of alcohol resulted in reaching a 99% yield of the ester after 45 min, while at the equimolar amount of reagents, only a 30% yield was achieved due to the equilibrium limitations. Nevertheless, a higher concentration of relatively hydrophilic *n*-butanol led to an irreversible decrease in the activity of the biocatalyst, otherwise the reaction rate should have been higher. Furthermore, the experiments without any additional solvent at the higher amount of liquid reagents (1 mL of *n*-butanol or 1 mL of levulinic acid) confirmed the negative influence of a high concentration of those reagents on the activity of the biocatalyst.

**Figure 6:**
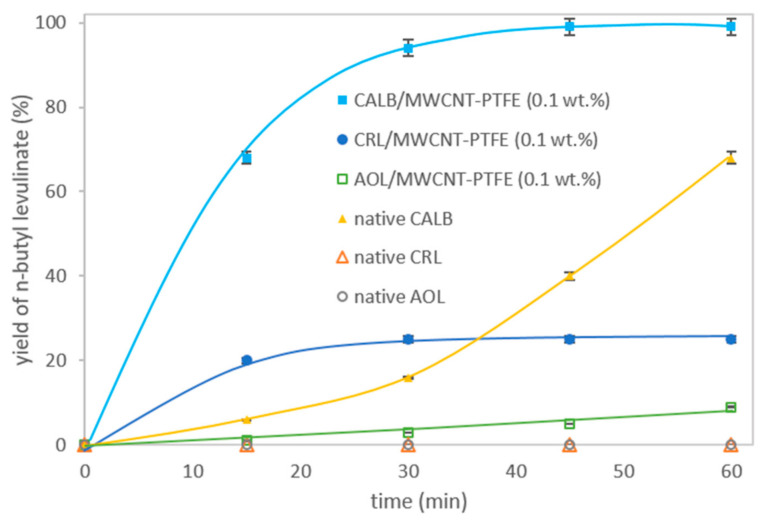
Comparison of native and immobilized lipases in LA esterification. Reaction conditions: LA (0.117 g, 1 mmol); *n*-butanol (0.148 g, 2 mmol); biocatalyst lipase/MWCNT-PTFE (0.10 wt.%) (150 mg) or native lipase 50 mg; cyclohexane (1 mL); 20 °C; 250 rpm. CRL: lipase from *Candida rugosa*; AOL: lipase from *Aspergillus oryzae.*

CALB is one of the most stable lipases due to a relatively small lid covering the active centre and the relatively slight tendency to deactivation via agglomeration caused by the interfacial activation with the further formation of dimer [50]. Nevertheless, for comparative purposes, the application of other lipases (lipase from *Candida rugosa* (CRL) and lipase from *Aspergillus oryzae* (AOL)) was also taken into the consideration. Table 2 showed a content of lipases after immobilization on the support contained 0.10 wt.% of PTFE and the activity of the biocatalysts in the same model reaction. The results of the experiments performed with biocatalysts in their native and immobilized forms were collected in Figure 6. Importantly, the experiments further confirmed the unique increase in the activity of CALB (up to 153,000 U∙L∙kg^−1^ of specific activity after 15 min, Table 2) after the immobilization on the MWCNT-PTFE hydrophobic support as well as a high stability even in its native form. Despite the application of native and immobilized forms of CRL and AOL, in each and every case the enzyme underwent fast deactivation. On the other hand, the specific activity of CRL in the model reaction reached 31,200 U∙L∙kg^−1^, which was yet higher than its native form. Lower activity of CRL and AOL might result from them having a larger lid than in CALB, thus the hydrophobicity of the PTFE-modified support might be too high for both of them. Thermogravimetric analysis (TGA) confirmed the lower amount of CRL and AOL adsorbed on the surface of the hybrid support, i.e., 15.6 and 7.0 wt.%, respectively.

**Figure 7:**
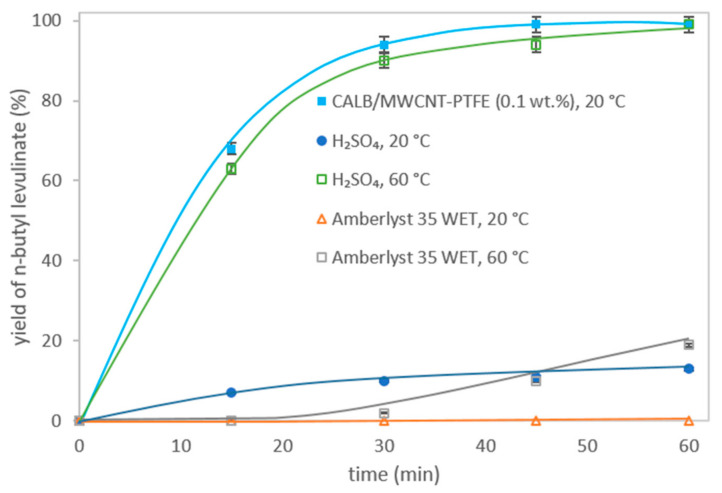
Comparison of various catalysts in LA esterification. Reaction conditions: LA (0.117 g, 1 mmol); *n*-butanol (0.148 g, 2 mmol); catalyst: biocatalyst CALB/MWCNT-PTFE (0.10 wt.%) (150 mg), CALB (50 mg), H_2_SO_4_ (7 mg), Amberlyst 35 WET (150 mg); cyclohexane (1 mL); 250 rpm.

Consequently, the activity of the CALB/MWCNT-PTFE (0.10 wt.%) biocatalyst was compared with sulfuric (VI) acid and Amberlyst 35 WET ion exchange macromolecular resin as standard acidic homogeneous and heterogenous catalysts, respectively. Figure 7 shows that the reaction with the immobilized biocatalyst was considerably faster and more efficient than the reaction with sulfuric (VI) acid for both processes conducted at 20 °C. The sulphuric acid was more active at 60 °C (94% yield after 45 min), with similar activity to the CALB/MWCNT-PTFE (0.1 wt.%) biocatalyst at 20 °C (99% yield after 45 min). The strongly acidic resin Amberlyst 35 WET showed a rather low activity (13% yield after 60 min). These experiments clearly confirmed the advantage of the elaborated biocatalytic system.

**Figure 8:**
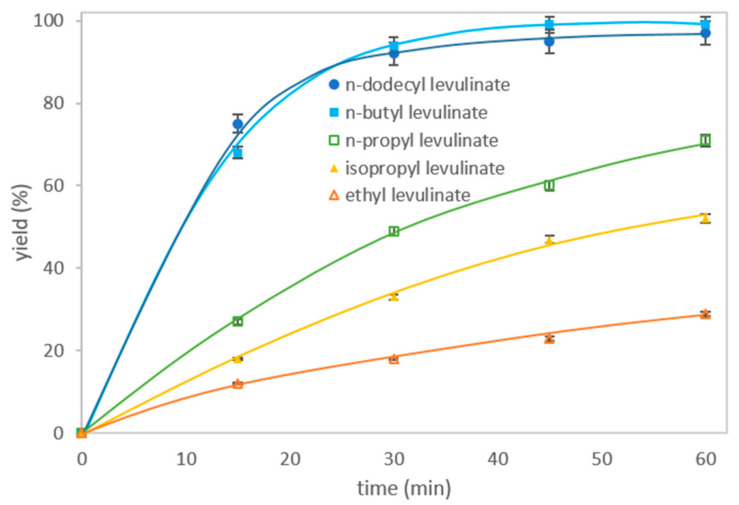
The effect of the alcohol on LA esterification. Reaction conditions: LA (0.117 g, 1 mmol); alcohol (2 mmol); biocatalyst CALB/MWCNT-PTFE (0.10 wt.%) (150 mg); cyclohexane (1 mL); 20 °C; 250 rpm.

In order to present the versatility of the so-developed method, the reactivity of other alcohols was studied. The results clearly show that the ester yield increased with the length of alkyl chain of the given alcohol (Figure 8). In the case of *n*-butanol and *n*-dodecanol, after 45 min, the yield of esters reached 99% and 95%, respectively. This is due to the fact that lipase B from *Candida antarctica* generally displays the highest activity in the reactions with linear and long- or medium-chain length substrates [51,52]. The use of short-chain alcohols, such as ethanol and *n*-propanol, with a strong nucleophilic character and higher polarity, resulted in the decline of yield of the corresponding esters (71% for ethyl levulinate and only 29% for *n*-propyl levulinate after 60 min). It is probably caused by the damaging of the enzyme structure, e.g., by denaturation in the presence of these alcohols. Comparing the course of the reaction with *n*-propanol and isopropanol, the esterification was slower for the latter which is due to the steric hindrance in the secondary alcohol.

**Figure 9:**
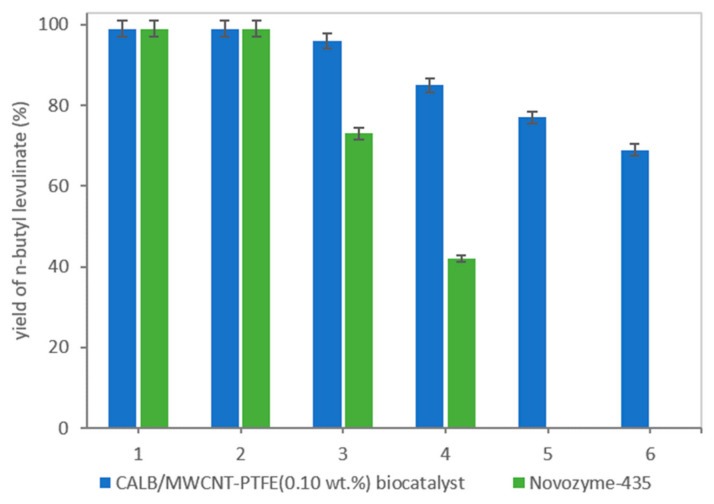
Catalyst recycling. Reaction conditions: LA (0.117 g, 1 mmol); *n*-butanol (0.148 g, 2 mmol); biocatalyst CALB/MWCNT-PTFE (0.10 wt.%) (150 mg); cyclohexane (1 mL); 45 min, 20 °C; 250 rpm.

Last but not least, the stability of the biocatalysts CALB/MWCNT-(0.1 wt.%)PTFE was studied. For this purpose, after the reaction, the biocatalyst was filtered off, washed with cyclohexane, dried under vacuum and then used for the next cycle of the reaction. The experiments were conducted using the two-fold molar ratio of LA to *n*-butanol at 20 °C, and the samples were analysed after 45 min. After each isolation of the catalyst from the reaction mixtures, a slight weight loss of the biocatalyst was observed (from 2% to 5%). Nevertheless, the results show that immobilization of CALB via adsorption on the hybrid carrier was indeed an effective method (Figure 9). Although this technique uses weak physical interactions between the lipase and the carrier, only negligible leaching of the catalyst was observed over the three cycles and the ester yield remained at 99% after 45 min, which was further confirmed via the Lowry’s technique (ESI, Chapter S5).

In the light of a lack of intensive enzyme leaching, even in a hydrophobic solvent, which usually facilitates a desorption of a lipase [53], the deactivation of the biocatalyst after three cycles might be caused by the adsorption of the substrates and/or product onto the active centres of lipase, limiting its availability. In addition, the presence of hydrophilic compounds in the reaction mixture and the generation of water during the process might have caused the faster deactivation of lipase due to formation of hydrophilic layers inside the structure of the biocatalyst [50]. Hence, additional operations can be used in the future to improve the stability of the biocatalyst, e.g., the coating of the biocatalyst using silicon which increases the mechanical stability or the cross-linking of a lipase within a matrix in order to eliminate leaching of the enzyme [53,54]. Critically, as revealed in three comparative experiments, it must be underlined that the developed biocatalytic system exhibited higher stability in the reaction conditions than the commercially available, benchmark catalyst Novozyme-435 (CALB immobilized on the Lewatit VP OC 1600). In addition, the kinetics of both processes was compared (Figure S5).

## 4. Conclusions

The hybridization of PTFE and MWCNTs was demonstrated as an excellent route for obtaining high-performance support for the non-covalent immobilization of lipase B from *Candida antarctica*. The application of the hybrid provided the significant increase in the activity and stability of the enzyme in the fully selective esterification of levulinic acid and selected alcohols. The unique hybrid MWCNT-PTFE (0.10 wt.%) emerged in fact as considerably more active and stable than the commercially available forms of CALB and conventional homogenous catalysts.

The performed studies, also concerning the optimization of the reaction conditions, proved that the proposed approach allowed for the obtainment of high yields and selectivity of the whole spectrum of levulinic acid esters. Due to mild conditions (room temperature, atmospheric pressure) and the application of the highly active biocatalyst, the developed method outperforms the so-far elaborated protocols, even typical acidic catalysts. One might hence sum up here that the proposed methodology opens new avenues in nanobiocatalysis.

## Figures and Tables

**Table 1:** The properties of multi-wall carbon nanotube/ polytetrafluoroethylene (MWCNT/PTFE) hybrid materials used in this work. CALB: *Candida antarctica.*

Support	SBET (m^2^/g)	Pore Volume (cm^3^/g)	PTFE Loading (wt.%)	CALB Loading (wt.%)	Immobilization Yield (%)
PTFE, grains	1.6	0.004	n/a	0.7	0.1
CheapTubes™ MWCNTs	89.0	0.490	n/a	15.7	4.2
MWCNT-PTFE (0.01 wt.%)	83.1	0.204	0.06	16.0	4.3
MWCNT-PTFE (0.10 wt.%)	80.3	0.211	0.13	22.5	6.0
MWCNT-PTFE (0.50 wt.%)	86.3	0.223	0.46	15.9	4.2
MWCNT-PTFE (1.00 wt.%)	84.2	0.222	0.89	13.3	3.5
MWCNT-PTFE (10.00 wt.%)	87.4	0.215	4.24	12.4	3.3
MWCNT-PTFE (20.00 wt.%)	77.1	0.208	10.56	11.7	3.1

**Table 2:** The properties of MWCNT/PTFE hybrid materials used in this work.

Support	Lipase Loading (wt. %) ^a^	Total Activity (U∙L) ^b^	Specific Activity (U∙L∙kg^−1^) ^b^
CALB/MWCNT-PTFE (0.10 wt.%)	22.5	45,333	153,000
CRL/MWCNT-PTFE (0.10 wt.%)	15.6	13,333	31,200
AOL/MWCNT-PTFE (0.10 wt.%)	7.0	667	700

^a^ Values determined via thermogravimetric analysis (ESI, Chapter S4). ^b^ Total and specific activities of the biocatalyst determined after 15 min of the esterification between LA and *n*-butanol (ESI, Chapter S4).

## Supplementary Materials

The following are available online at https://www.mdpi.com/1996-1944/14/6/1518/s1, Figure S1: Dark-field TEM image of MWCNT-PTFE (0.10 wt.%) support, Figure S2: Dark-field TEM image of CALB/MWCNT-PTFE (0.10 wt.%) biocatalyst, Table S1: EDS analysis of MWCNT-PTFE (0.10 wt.%) support and CALB/MWCNT-PTFE (0.10 wt.%) biocatalyst, Table S2: Temperature program of GC-FID analysis, Table S3: Retention time of products and the parameters of linear regression, Scheme S1: 1H NMR spectrum of ethyl levulinate, Scheme S2: 13C NMR spectrum of ethyl levulinate, Scheme S3: 1H NMR spectrum of n-propyl levulinate, Scheme S4: 13C NMR spectrum of n-propyl levulinate, Scheme S5: 1H NMR spectrum of isopropyl levulinate, Scheme S6: 13C NMR spectrum of isopropyl levulinate, Scheme S7: 1H NMR spectrum of n-butyl levulinate, Scheme S8: 13C NMR spectrum of n-butyl levulinate, Scheme S9: 1H NMR spectrum of n-dodecyl levulinate, Scheme S10: 13C NMR spectrum of n-dodecyl levulinate, Scheme S11: The TG curve of CRL solution. The lipase content was estimated in a range of 180–400 °C, Scheme S12: The TG curve of CRL/MWCNT-PTFE (0.10 wt.%) biocatalyst. The lipase content was estimated in a range of 180–400 °C, Scheme S13: The TG curve of AOL. The lipase content in the commercial solution was estimated in a range of 180–400 °C, Scheme S14: The TG curve of AOL/MWCNT-PTFE (0.10 wt.%) biocatalyst. The lipase content was determined in a range of 180–400 °C, Figure S3. Raman spectra for MWCNT, PTFE, CALB/MWCNT-PTFE, Figure S4: Characterization of the immobilization process, Figure S5: Comparison of the course of the reaction of a model reaction with the use of biocatalyst CALB/MWCNT-PTFE (0.10 wt.%) and Novozyme-435.
