# Supplementary material for: PTFE-Carbon Nanotubes and Lipase B from Candida antarctica—Long-Lasting Marriage for Ultra-Fast and Fully Selective Synthesis of Levulinate Esters
Source: Materials (Basel). 2021 Mar 19;14(6):1518. doi: 10.3390/ma14061518 (PMC8003637; doi:10.3390/ma14061518)
Supplement: Supplementary file 1 [file materials-14-01518-s001.pdf]

Electronic Supplementary Information

PTFE-carbon nanotubes and lipase B from *Candida antarctica* – long-lasting marriage for ultra-fast and fully selective synthesis of levulinate esters

Anna Szelwicka [1]*, Agnieszka Siewniak* [1]*, Anna Kolanowska* [2]*, Sławomir Boncel* [2*] *and Anna Chrobok* [1*]

[1] Department of Organic Chemical Technology and Petrochemistry, Silesian University of Technology, Krzywoustego 4, 44-100 Gliwice, Poland

[2] Department of Organic Chemistry, Bioorganic Chemistry and Biotechnology, Silesian University of Technology, Krzywoustego 4, 44-100 Gliwice, Poland

Chapter S1. Characterization of the hybrid supports

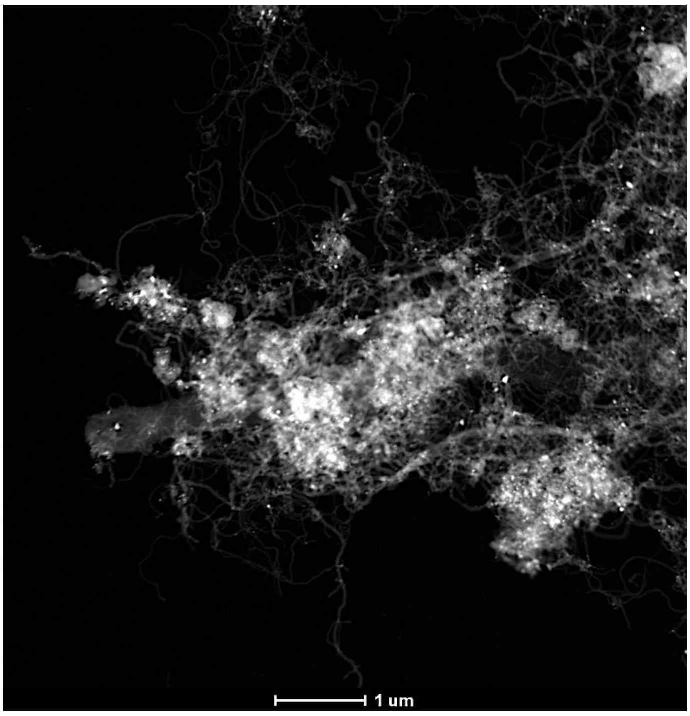

Figure S1. Dark-field TEM image of MWCNT-PTFE(0.10 wt.%) support

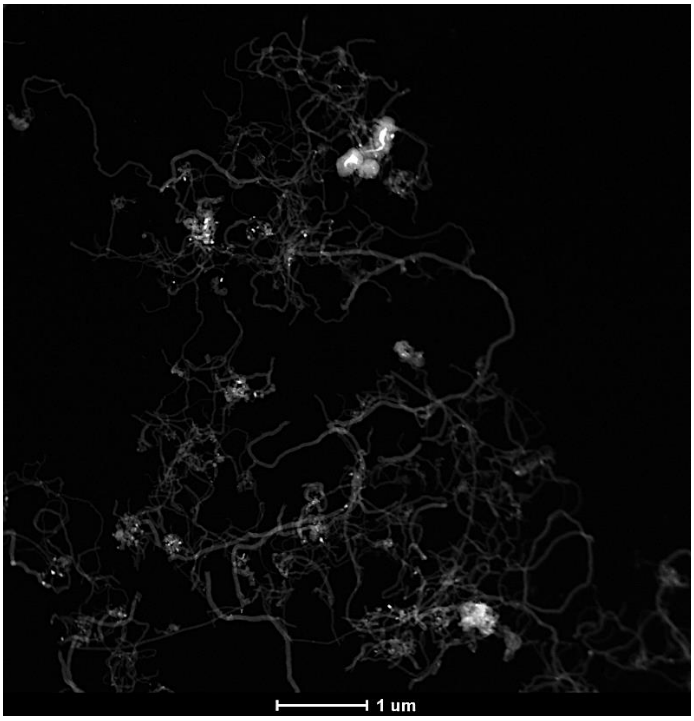

Figure S2. Dark-field TEM image of CALB/MWCNT-PTFE(0.10 wt.%) biocatalyst

Dark-field TEM images allowed to notice the differences between the hybrid MWCNT-PTFE(0.10 wt.%) support and CALB/MWCNT-PTFE(0.10 wt.%) biocatalyst. After immobilization of the lipase CALB, localization of Teflon species were found as less visible. In addition, in Table S2, the data from EDS analysis of both materials, eventually indicating a presence of the lipase (S and N atoms), were summed up.

Table S1. EDS analysis of MWCNT-PTFE(0.10 wt.%) support and CALB/MWCNT-PTFE(0.10 wt.%) biocatalyst.[1]

Atom	Mass content of atom in the material (%)	
	MWCNT-PTFE(0.10 wt.%) support	CALB/MWCNT-PTFE(0.10 wt.%) biocatalyst
C	95.16	98.86
O	4.74	0.44
N	-	0.38
F	0.10	0.16
S	-	0.16

Chapter S2. GC-FID analysis

Table S2. Temperature program of GC-FID analysis.

Initial temperature (°C)	Increasing rate of the temperature (°C/min)	Holding time (min)
80	-	2
80	40	0
200	20	0
280	-	5

Table S3**.** Retention time of products and the parameters of linear regression.

Compound	Retention time (min)	Linear regression equation	Correlation coefficient (R^2)
ethyl levulinate	4.165	y=0.4950x-0.1556	0.9997
n-propyl levulinate	4.705	y=0.4554x-0.1254	0.9989
iso-propyl levulinate	4.375	y=0.5414x-0.0638	0.9987
n-butyl levulinate	5.193	y=0.8451x-0.2032	0.9997
n-dodecyl levulinate	8.610	y=0.9964x-0.1061	0.9997

Chapter S3. ^{1}H and ^{13}C NMR spectra of products

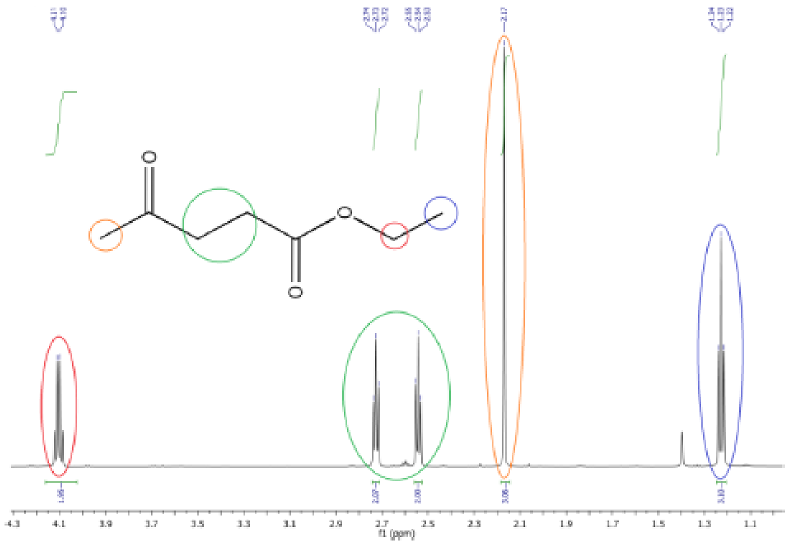

Scheme S1. ^{1}H NMR spectrum of ethyl levulinate.

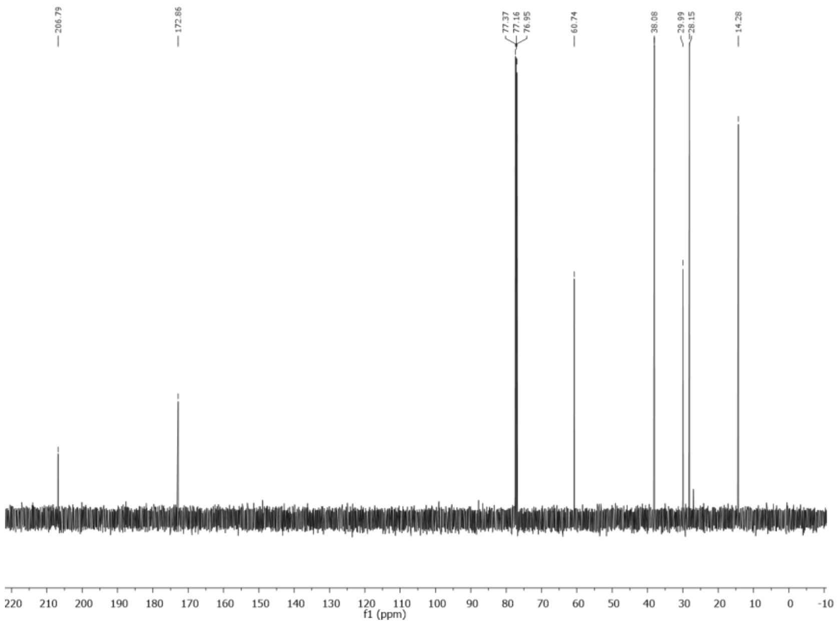

Scheme S2. ^{13}C NMR spectrum of ethyl levulinate.

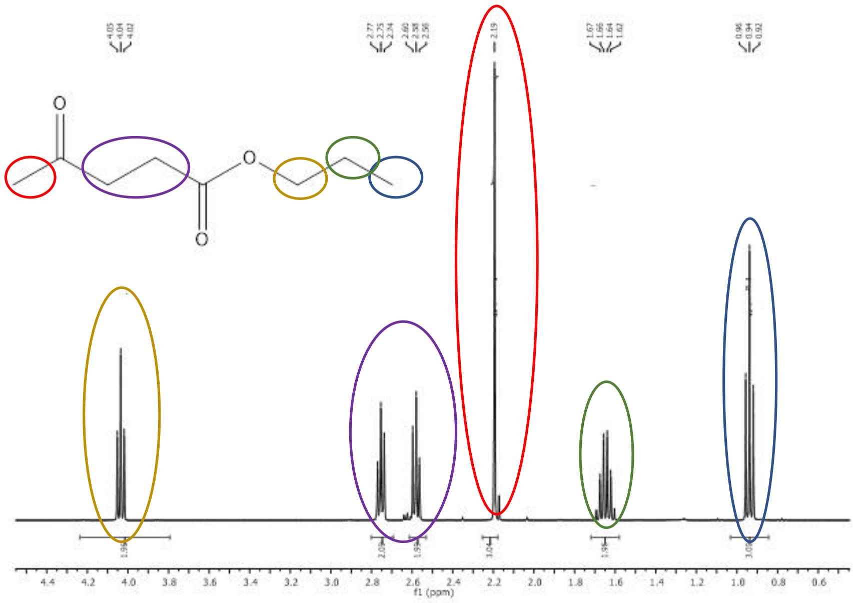

Scheme S3. ^{1}H NMR spectrum of *n*-propyl levulinate.

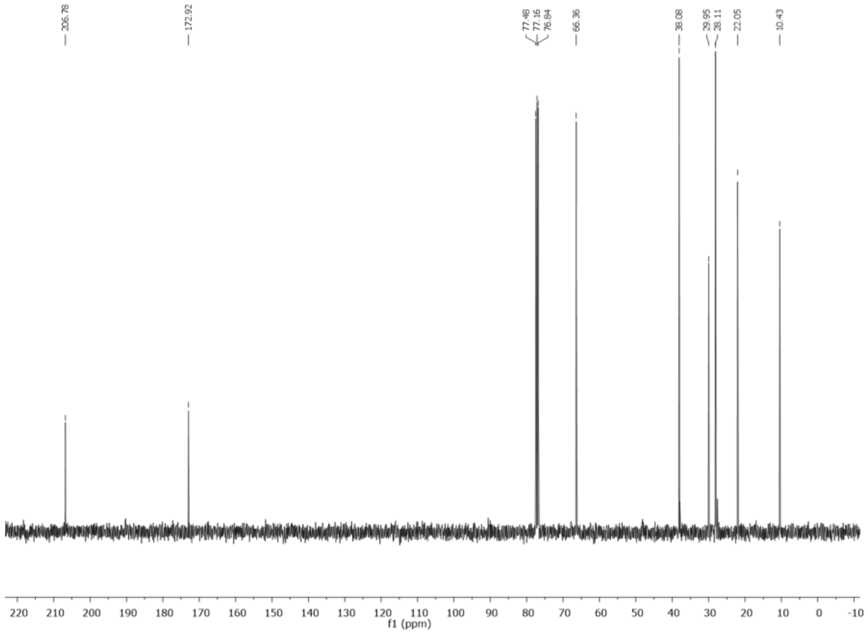

Scheme S4. ^{13}C NMR spectrum of *n*-propyl levulinate.

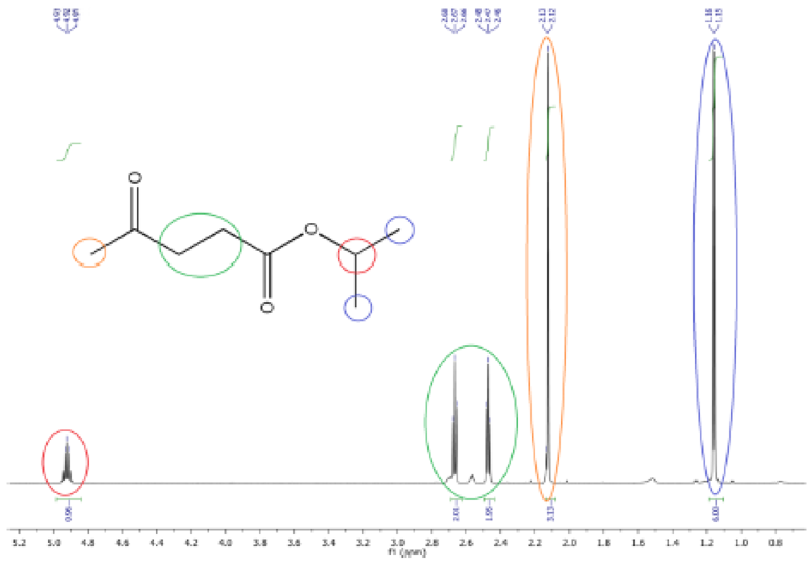

Scheme S5. ^{1}H NMR spectrum of isopropyl levulinate.

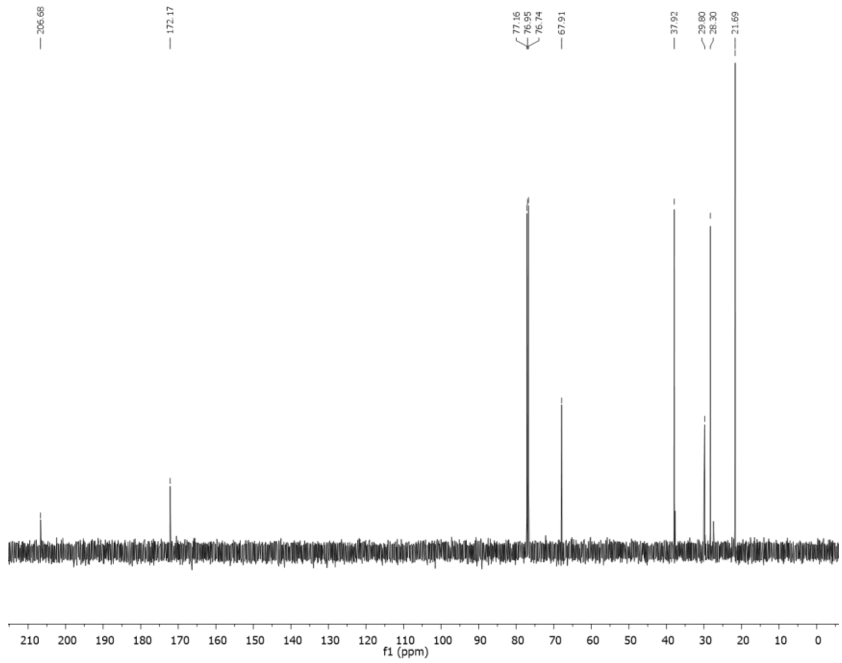

Scheme S6. ^{13}C NMR spectrum of isopropyl levulinate.

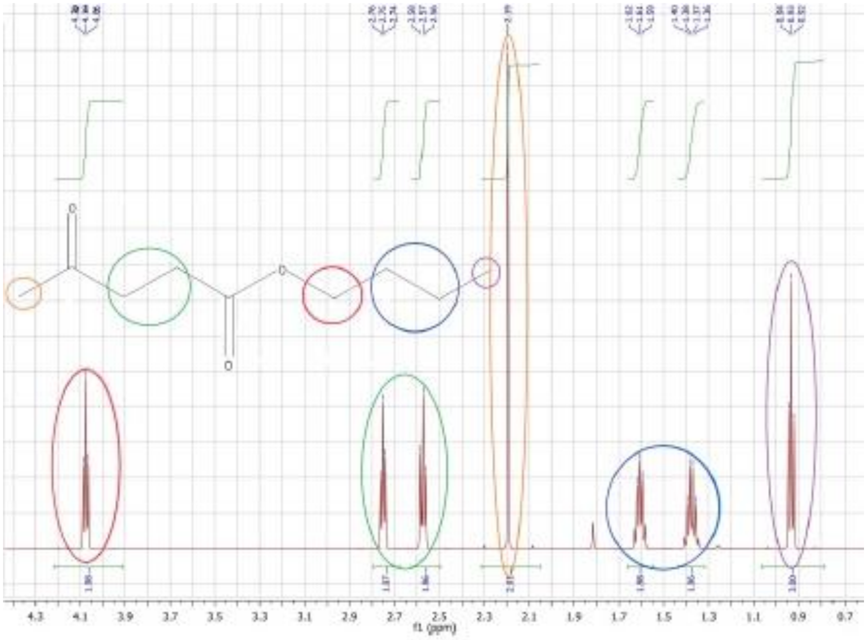

Scheme S7. ^{1}H NMR spectrum of *n*-butyl levulinate.

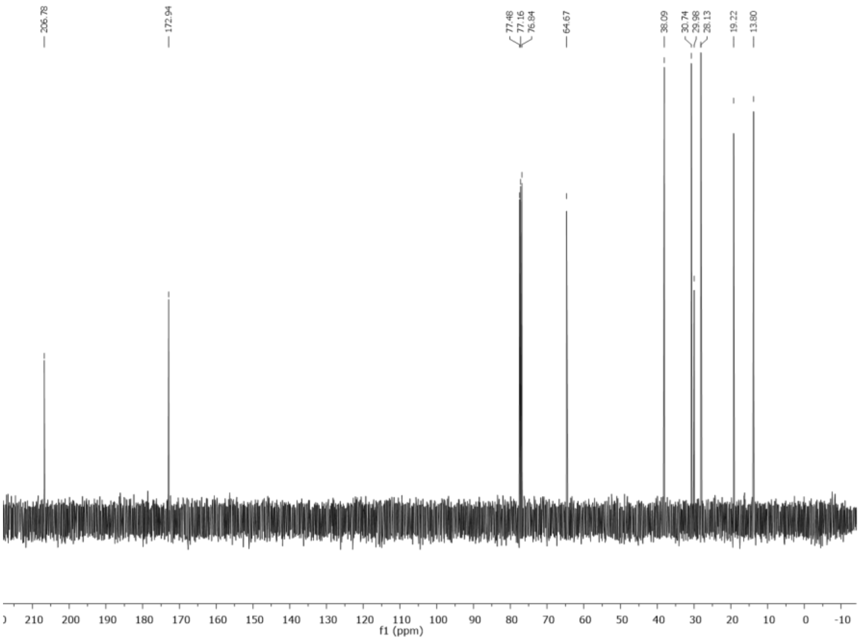

Scheme S8. ^{13}C NMR spectrum of *n*-butyl levulinate.

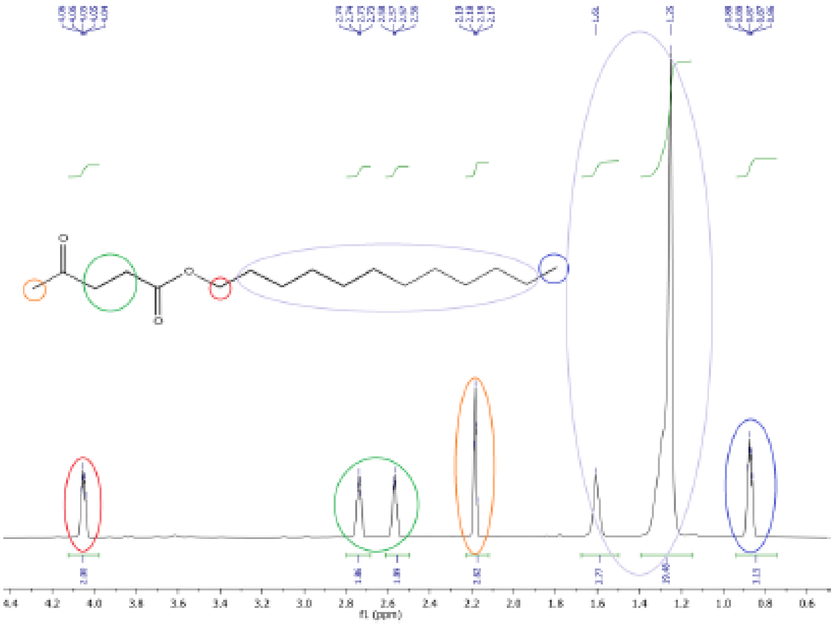

Scheme S9. ^{1}H NMR spectrum of *n*-dodecyl levulinate.

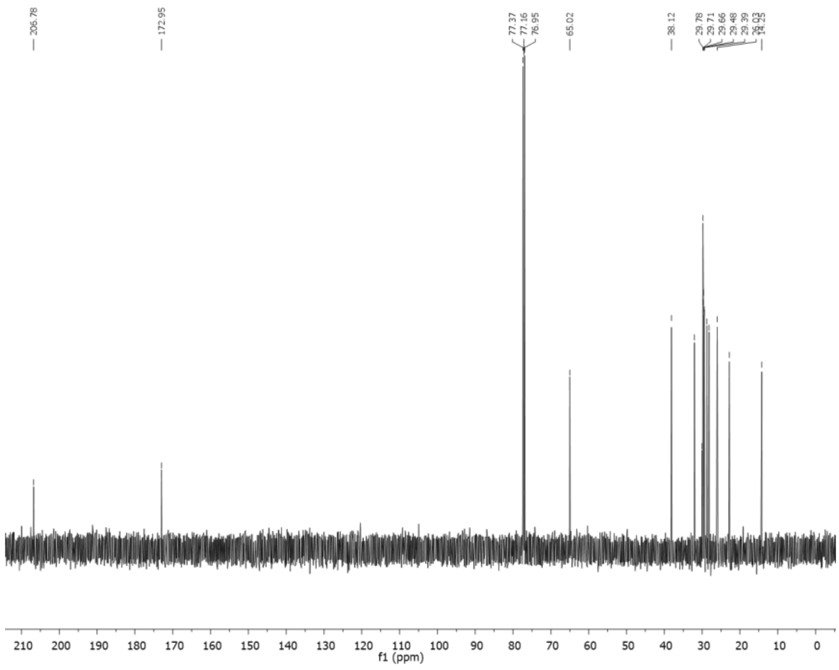

Scheme S10. ^{13}C NMR spectrum of *n*-dodecyl levulinate.

Chapter S4. TG curves of biocatalysts and calculations toward lipases' activities

The TG parameters of standards (PTFE, hybrid supports, lipase) and the CALB/MWCNT-PTFE(0.10 wt.%) biocatalyst were presented in our previous work[1].

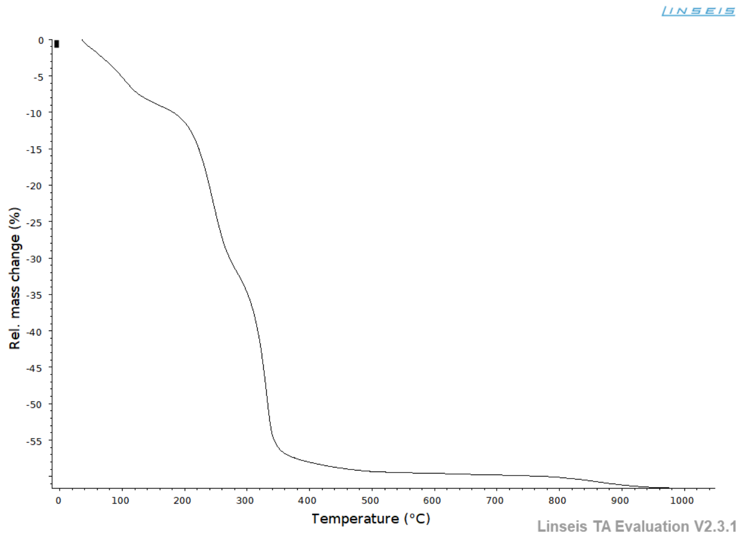

Scheme S11. The TG curve of CRL solution. The lipase content was estimated in a range of 180-400 °C.

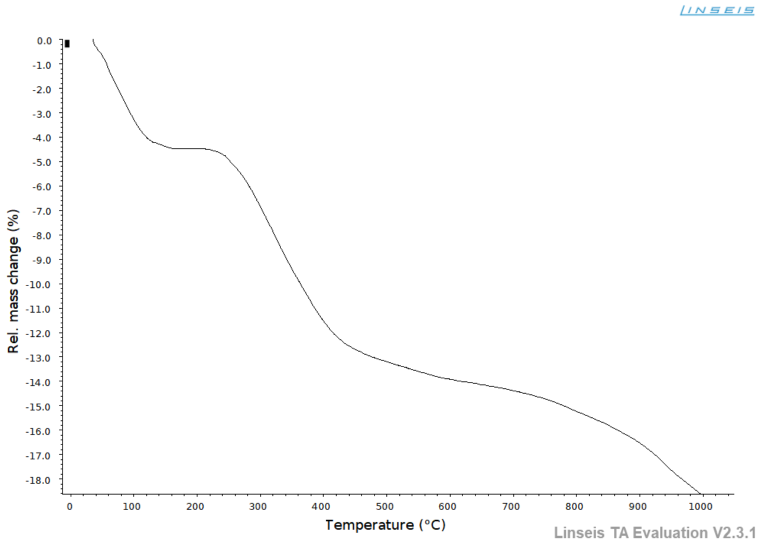

Scheme S12. The TG curve of CRL/MWCNT-PTFE(0.10 wt.%) biocatalyst. The lipase content was estimated in a range of 180-400 °C.

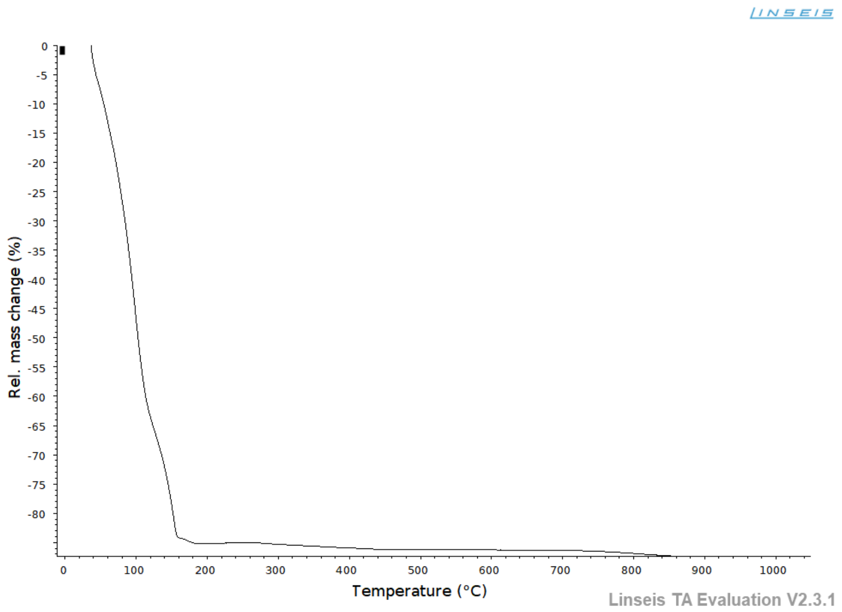

Scheme S13. The TG curve of AOL. The lipase content in the commercial solution was estimated in a range of 180-400 °C.

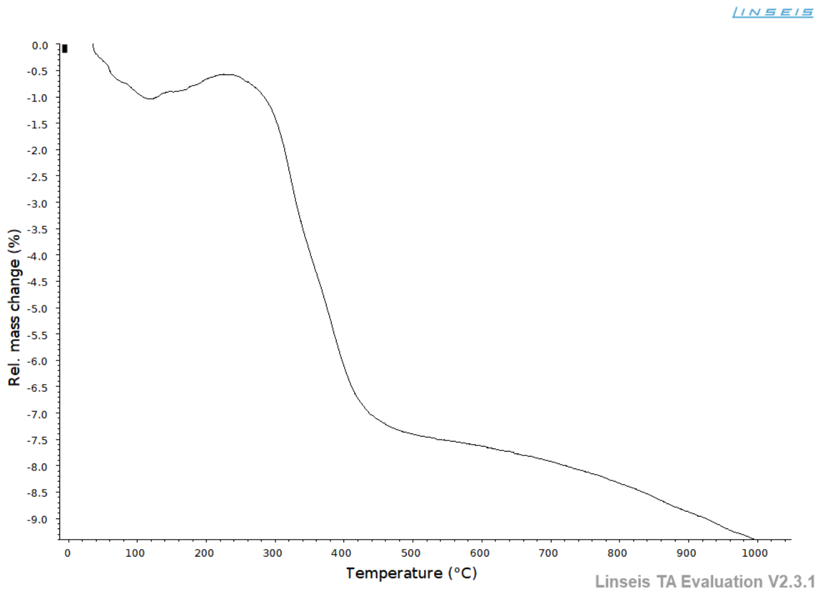

Scheme S14. The TG curve of AOL/MWCNT-PTFE(0.10 wt.%) biocatalyst. The lipase content was determined in a range of 180-400 °C.

Lipases' activity was calculated as follows:

1. Mass of lipase was determined based on the wt.% of the protein adsorbed onto support (TGA analysis)
2. Reaction volume after 15 min was determined from the density of reactants and solvents (assuming appropriate conversion of substrate, yield of the product and lack of by-products)
3. Yield of the product after 15 min allowed to determine the mole of product obtained and the conversion of substrate
4. Total activity was determined from the definition as an amount of enzyme, which catalyzes the conversion of one μmol of substrate, after calculating how many mole of product was synthesized average per minute. Total activity was presented in enzyme units (U = μmol/s) times the volume
5. Specific activity was determined based on the value of total activity and the amount of lipase in the reaction mixture, showed as U·L/kg.

Chapter S5. The stability measurements of CALB

In order to confirm the lack of protein in the filtrate, the Lowry's protein detection method was selected. The experiments were carried out as follows:

Lowry's protein detection method

Content of the lipase in the filtrate was calculated *via* Lowry's method of protein detection using a UV-VIS technique. Jasco V-650 spectrophotometer was used at room temperature in aqueous solution. Calculations were performed based on the value of the absorbance at wavelength λ=670 nm. This technique confirmed the protein was below the detection limit in the filtrate after all reaction cycles.

Preparation of the calibration curve for Lowry's protein detection method

To the 25 mL flask an aqueous solution of 3-30 μL/mL of *Candida antarctica* lipase B was introduced. The samples to obtain the calibration curve were prepared *via* mixing of 1 mL of protein solution with 5 mL of 2% solution of Na_2CO_3 in 0.1 M aqueous solution of NaOH. 10 min after mixing, a 0.5 mL of Folin-Ciocalteu reagent was added and the absorbance at wavelength λ=670 nm was measured after next 30 min. Each measurement was repeated twice and the calibration curve with R^2 equal 0.979 was achieved as a consequence.

Chapter S6. Raman spectra for MWCNT, PTFE, CALB/MWCNT-PTFE

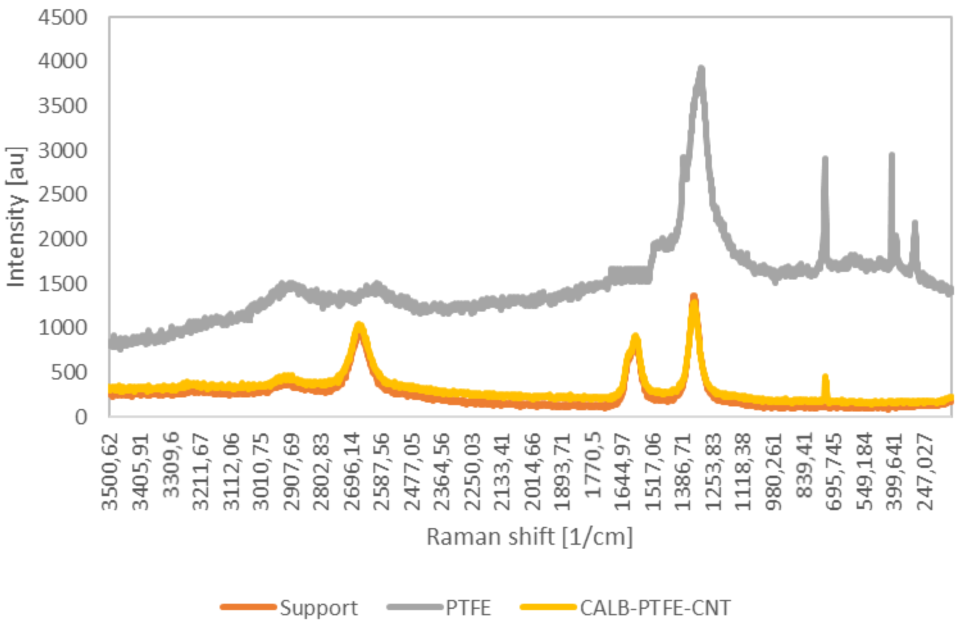

Figure S3. Raman spectra for MWCNT, PTFE, CALB/MWCNT-PTFE.

Chapter S7. Characterization of the immobilization process

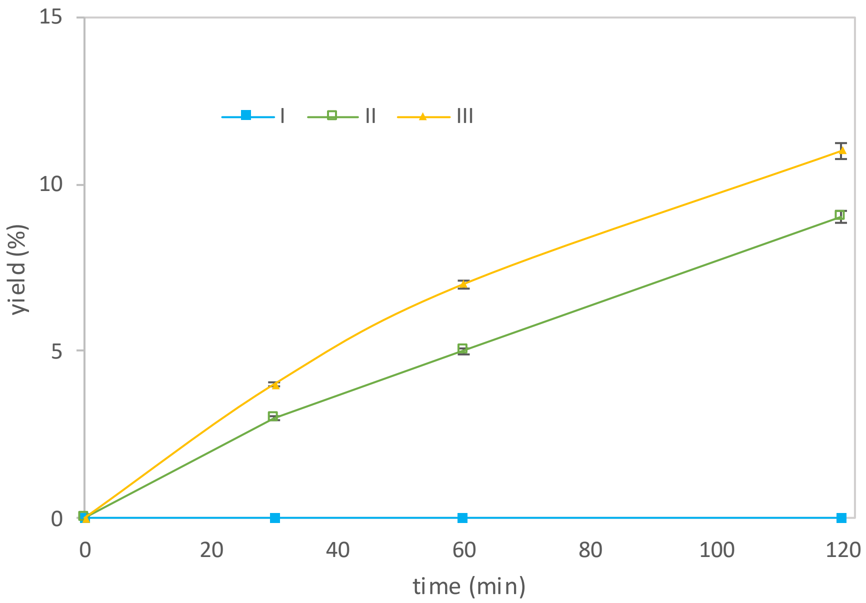

Figure S4. Comparison of the course of: (I) reaction with the filtrate after synthesis of CALB/MWCNT-PTFE (0.1 wt.%) biocatalyst in an amount equal to the amount of CALB immobilized on the biocatalyst; (II) reaction with equivalent amount of aqueous solution of CALB after immobilization without support; and as a reference (III) reaction with fresh solution of CALB diluted by deionized water to the same amount as in experiments (I) and (II). Reaction conditions: LA (0.117 g, 1 mmol); *n*-butanol (2 mmol); cyclohexane (1 mL); 0.27 mL of CALB solution (0.125 g CALB/mL); 20°C; 250 rpm.

Chapter S8. Comparison of the course of the reaction of a model reaction with the use of biocatalyst CALB/MWCNT-PTFE(0.10 wt.%) and Novozyme-435.

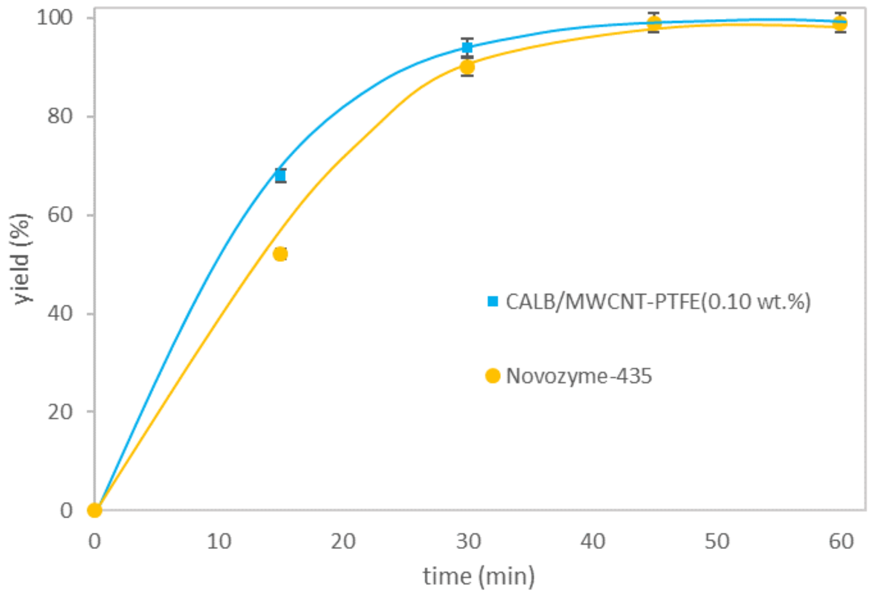

Figure S5. Comparison of the course of the reaction of a model reaction with the use of biocatalyst CALB/MWCNT-PTFE(0.10 wt.%) and Novozyme-435. Reaction conditions: LA (0.117 g, 1 mmol); *n*-butanol (2 mmol); biocatalyst CALB/MWCNT-PTFE(0.10 wt.%) or Novozyme-435 (150 mg); cyclohexane (1 mL); 20°C; 250 rpm.

Chapter S9. References

[1] A. Szelwicka, A. Kolanowska, P. Latos, S. Jurczyk, S. Boncel and A. Chrobok, *Catal. Sci. Technol.*, 2020, **10**, 3255.
